# Arthroscopic Implantation of Minced Cartilage From the *Os Trigonum* and Posterolateral Process of the Talus: Single‐Stage Cell‐Based Treatment of Osteochondral Lesion of the Talus

**DOI:** 10.1002/atn2.70096

**Published:** 2026-06-10

**Authors:** Hélder Pereira, Takuji Yokoe

**Affiliations:** ^1^ Orthopaedic Department Centro Hospitalar Póvoa de Varzim Vila do Conde Portugal; ^2^ Life and Health Sciences Research Institute (ICVS), School of Medicine University of Minho Braga Portugal; ^3^ ICVS/3B's ‐ PT Government Associate Laboratory Braga, Guimarães Portugal; ^4^ Ripoll y De Prado Sports Clinic: FIFA Medical Centre of Excellence Murcia, Madrid Spain; ^5^ Division of Orthopaedic Surgery, Department of Medicine of Sensory and Motor Organs, Faculty of Medicine University of Miyazaki Miyazaki Japan

## Abstract

The best surgical treatment of osteochondral lesion of the talus (OLT) remains controversial. Among the available surgical procedures for OLTs, implantation of the minced cartilage from the talar lesion is a promising cell‐based technique. However, surgeons may experience difficulties when enough volume and quality of the cartilage cannot be obtained from the talar lesion. In addition, some patients such as athletes simultaneously suffer from both OLT and posterior ankle impingement syndrome. This article describes the possibility of implantation of minced cartilage from the *Os Trigonum* and/or posterolateral process of the talus to treat OLTs. Furthermore, this technique can simultaneously treat both the OLT and posterior ankle impingement syndrome in a single stage with a minimal risk of donor‐site morbidity. Considering the low morbidity related to harvesting even asymptomatic *Os Trigonum* and/or posterolateral process of the talus, this technique might be a valid option for chondral lesions in other joints.

## Graphical abstract

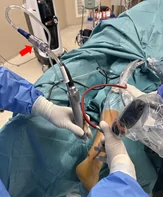

**VIDEO 1:** This video provides a step‐by‐step description of a classical indication for the use of arthroscopic implantation of minced cartilage harvested from the *Os Trigonum*. It concerns the surgical treatment of the right ankle of a 28‐year‐old male, with a symptomatic anterolateral osteochondral lesion of the talus (OLT) in zones 3/6, combined with posterior impingement syndrome and chronic lateral ankle instability. The procedure begins in prone position for treatment of posterior impingement and harvesting minced cartilage from the *Os Trigonum*. After closure of the 2 posterior portals, the patient is changed to the supine position. An anterior 2‐portal ankle arthroscopy is performed. Synovectomy and removal of soft tissue/bony impingement may be performed. The OLT is debrided. It is possible to observe the degenerative aspect of the overlying cartilage (as opposed to the healthy cartilage harvested from the posterior compartment). The footprint area of the distal fibula (insertion of the anterior tibial‐fibular ligament [ATFL]) is debrided to bleeding bone, and 2 all‐soft tissue anchors are inserted. Afterward, by means of using a suture passer (or an 18‐gauge needle charged with a suture to be used as a shuttle), the remnant of the ATFL is grasped and left alone, prepared for final tension. In this case, tensioning of ATFL repair is performed at the last gesture in order to avoid complication possibly related to manipulation during the final treatment of OLT. At this point we change to dry arthroscopy, the OLT bed is dried by using a tissue absorber. The minced cartilage enhanced with autologous platelet‐derived growth factors (platelet‐rich plasma) is delivered to the defect by using a dedicated applier. Finally, a combination of platelet‐rich plasma and thrombin is put over the minced cartilage in dry conditions, and a period of 5 min to achieve minimal stabilization is performed. Now, tension is given to the sutures to finalize the ATFL repair and anterior portals are closed. Video content can be viewed at https://doi.org/10.1002/ant2.70096.

Osteochondral lesion of the talus (OLT) is a combined lesion of the subchondral bone and its overlying cartilage that often occurs following ankle trauma such as ankle sprains and fractures.^1^, ^2^ When nonsurgical treatment fails to resolve the patients’ symptoms, surgical treatment is generally considered.^3^ Although a great number of surgical procedures have been reported, no superior surgical procedure to other procedures exists for both primary and secondary OLTs.^4^, ^5^, ^6^, ^7^ Among available surgical techniques for OLTs, autologous minced cartilage implantation using the cartilage of the talar lesion is a promising cell‐based, single‐stage technique.^8^, ^9^ However, there is a lack of studies and evidence on the efficacy of application of this technique to OLTs, although there are several studies regarding knee osteochondral lesions.^10^, ^11^, ^12^ One of the critical limitations of this technique is the limited source of available autologous cartilage once the applied cartilage is harvested from the lesion cartilage, and iatrogenic chondral damage must be avoided. It has been reported that cartilage from the *Os Trigonum* or posterolateral process of the talus shows characteristics of the viable cartilage cells and can be available as a source of applied cartilage for cell‐based and tissue‐engineering treatment of chondral pathologies.^13^ The overall prevalence of the *Os Trigonum* from computed tomography and magnetic resonance imaging studies ranges from 15% to 25% in the general population while posterior talar process with any size or shape is a part of normal anatomy.^14^ Therefore, cartilage of the *Os Trigonum* and posterolateral process of the talus can be a source of viable cartilage when performing implantation of the minced cartilage to OLTs. In addition, some patients such as athletes sometimes suffer from both OLT and posterior ankle impingement syndrome (PAIS) due to *Os Trigonum* or prominent posterolateral process of the talus (Stieda's process). Several authors have reported favorable clinical outcomes following simultaneous arthroscopic treatment of both the OLT and PAIS.^15^, ^16^ The aim of this technical note is to describe the implantation of minced cartilage from the *Os Trigonum* and/or posterolateral process of the talus for the treatment of OLTs.

## SURGICAL TECHNIQUE

### Indications and Contraindications of the Presented Technique

The indications and contraindications of the presented technique are summarized in Table 1. Patients who suffer from both OLT and symptomatic *Os Trigonum* are the best indications of the present technique. However, those with asymptomatic *Os Trigonum* are also indicated if they consent to this surgical procedure.

**TABLE 1:** Indications and Contraindications of Implantation of Minced Cartilage From *Os Trigonum* to Osteochondral Lesion of the Talus

Indications
Osteochondral lesion of the talus and posterior ankle impingement syndrome (with and without *Os Trigonum*)
Osteochondral lesion of the talus and asymptomatic *Os Trigonum* (when patients consent to this surgical procedure)
Also possible application to other joints such as the knee joint

Contraindications
Patients who do not consent to harvesting cartilage from the *Os Trigonum* or posterolateral talar process

### Hindfoot Arthroscopic Evaluation and Excision of the *Os Trigonum*

**FIGURE 1:**
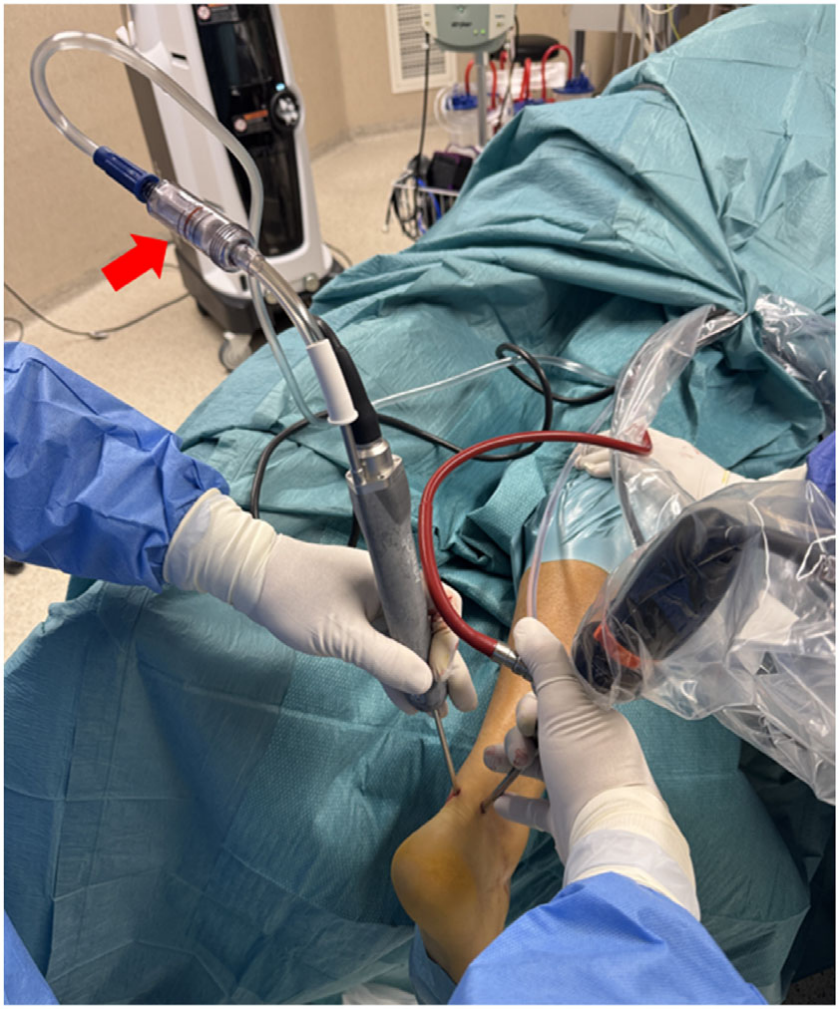
Harvesting hyaline cartilage from the *Os Trigonum* and posterolateral process of the talus under hindfoot arthroscopy (right foot). Hindfoot arthroscopy is performed with posteromedial and posterolateral portals. The cartilage is collected using an arthroscopic shaver that is connected to a collecting device (Graft‐Net, Arthrex, Naples, FL) (red arrow).

**FIGURE 2:**
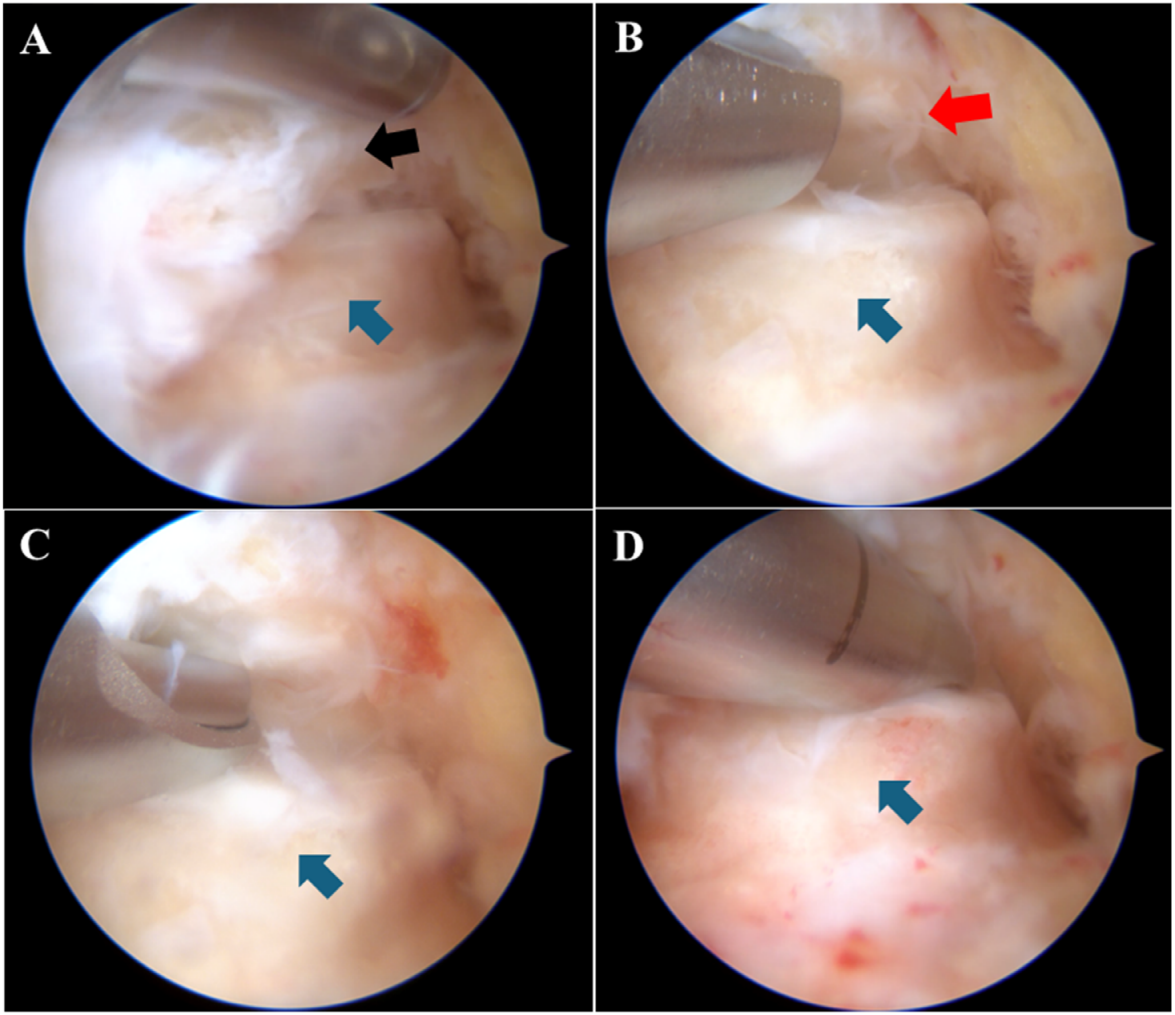
Hindfoot arthroscopic pictures via posterolateral portal (right foot). (A) Identifying the *Os Trigonum* (black arrow). (B) In the presence of *Os Trigonum*, the synchondrosis is detected (red arrow) and the *Os Trigonum* is separated from the talus. (C) Harvesting hyaline cartilage from the underlying surface of the *Os Trigonum*. (D) Harvesting hyaline cartilage from the calcaneus (blue arrow) while preserving the functional surface of the subtalar joint.

**FIGURE 3:**
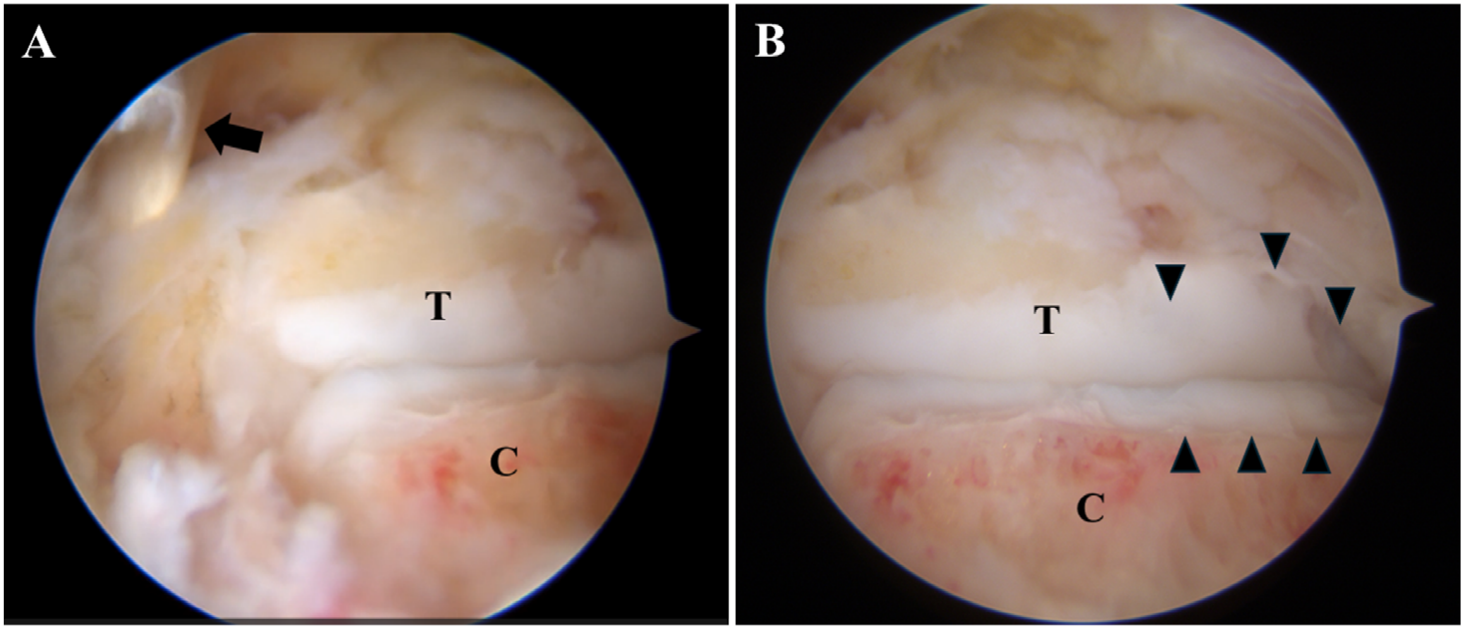
Hindfoot arthroscopic pictures via posterolateral portal (right foot). (A) After excision of the *Os Trigonum*. Flexor hallux longus tendon is indicated by a black arrow. (B) After harvesting hyaline cartilage from the posterolateral process of the talus and calcaneus. The arrowheads show the area where hyaline cartilage is harvested. (T, talus; C, calcaneus.)

**FIGURE 4:**
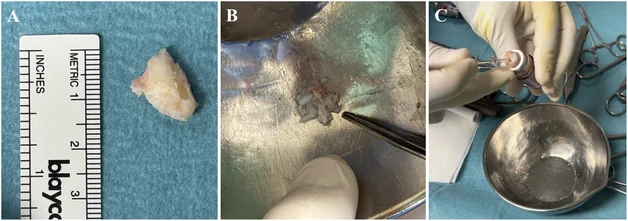
(A) A picture of the excised *Os Trigonum*. (B) Cartilage is harvested from the excised *Os Trigonum*. (C) The collected cartilage from the *Os Trigonum* or posterolateral process of the talus and calcaneus using a collecting device (Graft‐Net, Arthrex, Naples, FL).

The patient is placed in the prone position under general or spinal anesthesia following standard preparation of the affected lower extremity and administration of intravenous antibiotics. The tourniquet is applied over the operated thigh depending on the surgeon's discretion. Hindfoot arthroscopy is performed using posteromedial and posterolateral portals according to the technique described by van Dijk et al.^17^ After removing the proliferated synovitis around the *Os Trigonum* with an arthroscopic shaver and radiofrequency, the cartilage of the *Os Trigonum* or posterior talar process is collected using an arthroscopic shaver that is connected to a collecting device (Graft‐Net, AutoCart system, Arthrex, Naples, FL) (Figures 1 and 2A,B). The cartilage of the posterolateral process of the talus and, possibly, hyaline cartilage seen on the calcaneus surface not involving the functional subtalar joint (full range of subtalar motion assessed via arthroscopic evaluation) may also be harvested (Figures 2C,D and 3). It is mandatory not to make iatrogenic chondral damage into the subtalar joint. Then, the *Os Trigonum* is excised using a small chisel, as a whole piece. If present, the remaining cartilage of the *Os Trigonum* is additionally collected using a small nibbler (Figure 4). Thereafter, the course of flexor hallux longus tendon is arthroscopically assessed, and the absence of impingement with surrounding posterior compartment structures is confirmed.

### Arthroscopic Minced Cartilage Implantation to the OLT

**FIGURE 5:**
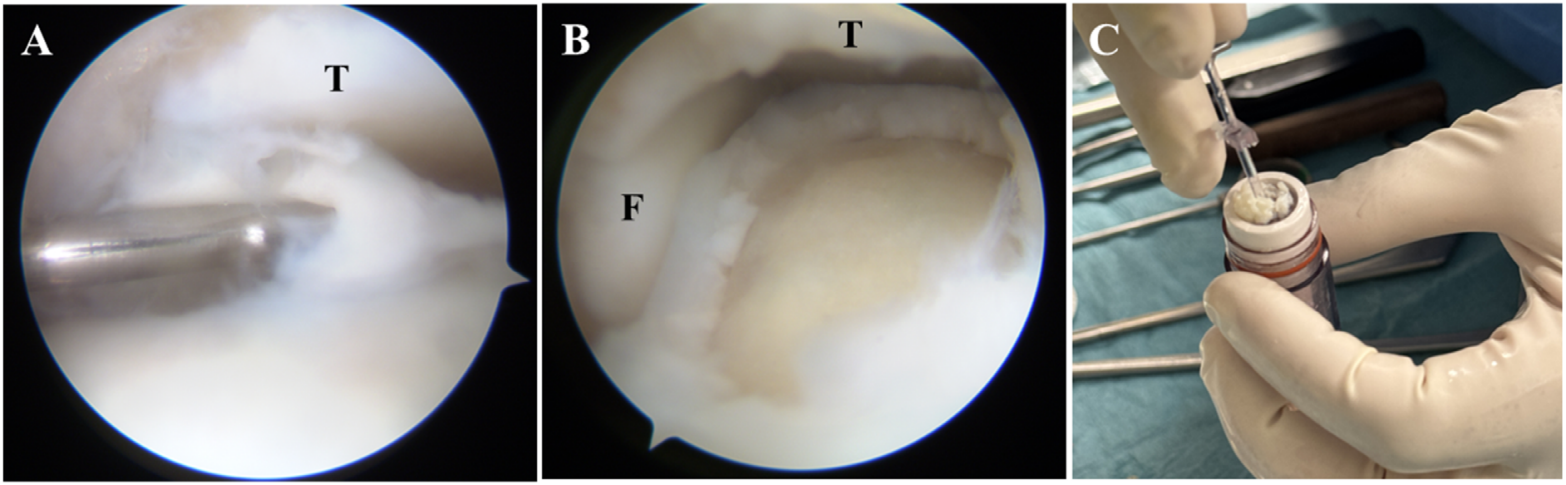
(A) Anterior ankle arthroscopic picture via anteromedial portal (right ankle). The lesion's cartilage is debrided and collected with an arthroscopic shaver that is connected to a collecting device (Graft‐Net, Arthrex, Naples, FL). (B) An anterior ankle arthroscopic picture via anterolateral portal. After completing the debridement of the talar lesion. (C) A picture of the collected lesion cartilage using a collecting device (Graft‐Net, Arthrex, Naples, FL) that is connected to an arthroscopic shaver. (F, fibula; T, tibia.)

**FIGURE 6:**
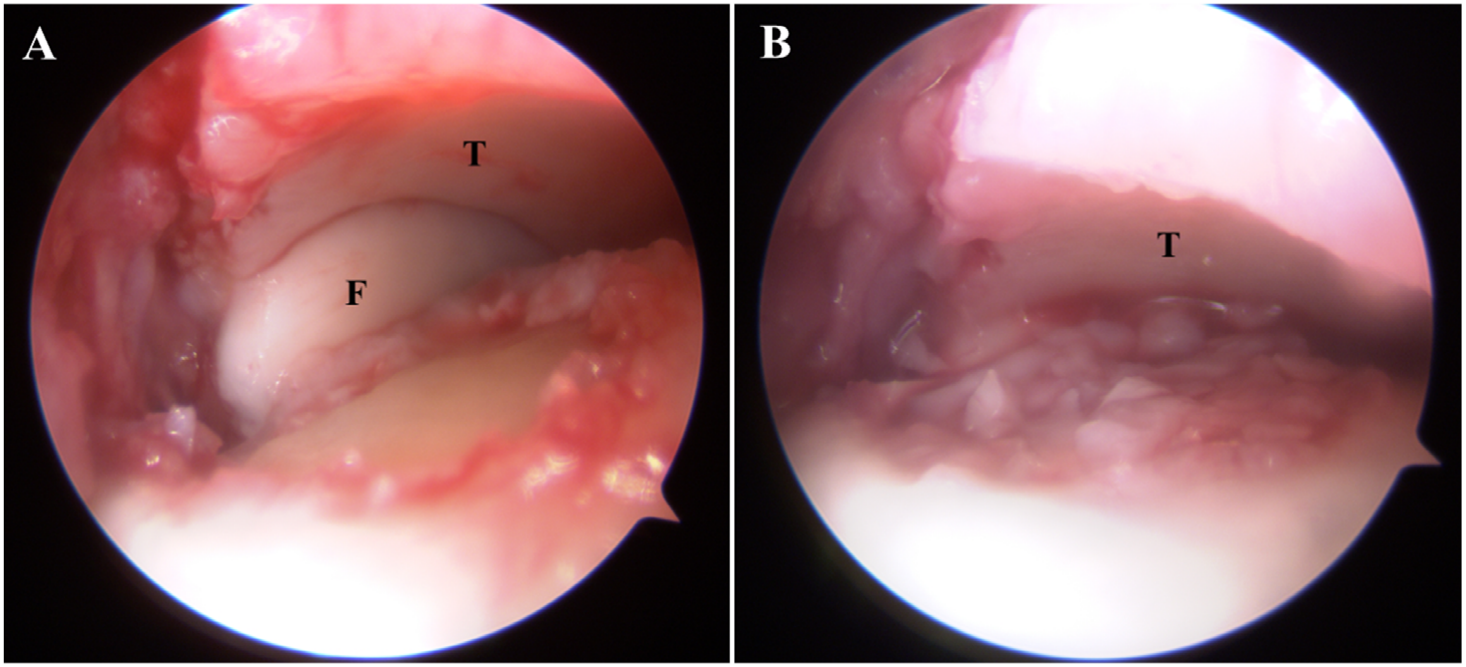
Anterior ankle arthroscopic pictures via anteromedial portal (right ankle). (A) Before implanting the minced cartilage/PRP paste to the talar lesion, the dry field of the talar defect needs to be maintained. (B) Placing the thrombin formed in a specific device (Thrombinator, Arthrex, Naples, FL) over the implanted cartilage/PRP paste to stabilize the implanted cartilage within the talar defect. (F, fibula; PRP, platelet‐rich plasma; T, tibia.)

The patient is placed in the supine position, and arthroscopic evaluation is performed using anteromedial and anterolateral portals. The principle of dorsiflexion, without ankle distraction, is applied during anterior ankle arthroscopy.^18^ This surgical technique can be applied to OLTs that are in any locations of the talus. Any concomitant ankle pathologies such as ligamentous instability, anterior ankle impingement, synovitis, or loose bodies should be addressed during the procedure. The cartilage of the OLT is collected using an arthroscopic shaver that is connected to a collecting device (Graft‐Net, Arthrex, Naples, FL) (Figure 5A,B). Thereafter, the collected minced cartilage from both the *Os Trigonum*, posterolateral process of the talus, and talar lesion is mixed with 3 or 4 platelet‐rich plasma (PRP) drops to create the minced cartilage/PRP paste according to the company's instructions^19^ (Figure 5C). In addition, PRP (3 mL) is inserted into a specific device (Thrombinator, Arthrex, Naples, FL) to produce thrombin that is used for stabilization of the implanted cartilage within the talar lesion. A dry field of the talar defect needs to be maintained using a suction device and surgical gauze, which can be used before implanting the minced cartilage/PRP paste to the lesion (Figure 6A). The minced cartilage/PRP paste is applied to the talar defect using a specific obturator device. It is important to make the surface of the filled defect the same height as the adjacent native cartilage. Finally, the thrombin formed in a specific device (Thrombinator, Arthrex, Naples, FL) is placed over the implanted minced cartilage/PRP paste. The thrombin combines with the PRP and generates fibrin, which coagulates quickly and fixes the implanted cartilage within the talar defect (Figure 6B). The surgical procedure is shown in Video 1.

### Postoperative Management

Postoperatively, the ankle is not immobilized, and ankle range of motion exercises are initiated as tolerated within 24 hours after surgery. Partial weight‐bearing is also allowed as tolerated immediately after surgery.

## DISCUSSION

Various kinds of surgical procedures for OLTs have been reported.^1^, ^20^ However, literature on ankle cartilage repair is still based on lower‐quality scientific evidence.^21^, ^22^ Minced cartilage implantation can be performed as a 1‐step procedure with promising short‐term surgical outcomes.^8^, ^9^ However, the lesion cartilage is used as a source of autograft in this technique. Although it has been reported that lesion cartilage from OLTs would be available as a source of autologous chondrocyte implantation,^23^ the lesion cartilage is not completely healthy and limited in volume for harvesting. Autologous osteochondral transplantation from the knee joint can provide favorable clinical outcomes for patients with OLT.^24^, ^25^ However, harvesting knee cartilage is associated with the risk of donor‐site morbidity.^26^ In addition, knee cartilage has different characteristics from ankle cartilage in terms of stiffness, proteoglycan synthesis and turnover.^27^, ^28^ The cartilage of the posterior superior calcaneal tuberosity can also be a possible donor site. However, Calder et al. reported that no hyaline cartilage was identified in the samples from the posterior superior calcaneal tuberosity.^29^

Correia et al. have reported that hyaline cartilage is detected in the *Os Trigonum* and posterolateral process of the talus.^13^ They also reported that isolation of the viable chondral cells is feasible from the *Os Trigonum* and posterolateral process of the talus.^10^ Some patients simultaneously suffer from both OLT and PAIS.^16^ Özer et al. reported that OLTs were detected in 18.3% of patients with PAIS by magnetic resonance imaging.^15^ The herein described surgical technique can not only simultaneously treat both the OLT and PAIS in a single stage but also use the cartilage from the *Os Trigonum* and/or posterolateral process of the talus to treat OLTs. In addition, this technique is associated with a minimal risk of donor‐site morbidity; thus, it is possible to envision that this source of autologous cell‐based therapy might also be applied to other joints (e.g., knee cartilage lesions).

However, clinical data following the presented technique is lacking and under research. Future clinical studies are needed to validate the mid‐ to long‐term efficacy and safety of the present technique. The advantages and disadvantages of this technique are shown in Table 2. The pearls and pitfalls of this technique are shown in Table 3.

**TABLE 2:** Advantages and Disadvantages of Implantation of Minced Cartilage from *Os Trigonum* to Osteochondral Lesion of the Talus

Advantages
Simultaneous and 1‐stage cell‐based treatment of posterior and anterior ankle pathologies
Low risk of donor‐site morbidity
Harvesting a greater amount of viable cartilage from other than the osteochondral lesion of the talus
Minimally invasive technique (all‐arthroscopically procedures)
Possible application to any location of the osteochondral lesion of the talus (also possible in other joints—e.g., knee)

Disadvantages
Depending on the quality of cartilage from the *Os Trigonum*, posterolateral talar process, and talar lesion
Scarce clinical data on the present technique
Requirement of dry field when implanting minced cartilage
Technically demanding

**TABLE 3:** Pearls and Pitfalls of Implantation of Minced Cartilage From *Os Trigonum* to Osteochondral Lesion of the Talus

Pearls
Surgeons need to be experienced in both posterior and anterior ankle arthroscopic skills to perform this technique
To excise the *Os Trigonum* arthroscopically, proliferated synovitis around the *Os Trigonum* should be cleaned up
Harvesting cartilage from the talar defect should not be excessive
The surface of the talar defect filled with minced cartilage/PRP paste should be flat to the adjacent native cartilage

Pitfalls
During hindfoot arthroscopy, iatrogenic injury to neurovascular bundles should be avoided using FHL as an anatomical reference
Functional joint surface of the subtalar joint is assessed arthroscopically and iatrogenic damage to the joint surface must be avoided
Before implanting the minced cartilage/PRP paste to the talar lesion, a completely dry bed in the lesion is needed

FHL, flexor hallux longus; PRP, platelet‐rich plasma.

## DISCLOSURES

The authors (H.P., T.Y.) declare that they have no known competing financial interests or personal relationships that could have appeared to influence the work reported in this article.
